# Influence of Ultrasound Treatment on Cavitation Erosion Resistance of AlSi7 Alloy

**DOI:** 10.3390/ma10030256

**Published:** 2017-03-03

**Authors:** Annalisa Pola, Lorenzo Montesano, Marialaura Tocci, Giovina Marina La Vecchia

**Affiliations:** DIMI-Mechanical and Industrial Engineering Department, Via Branze, 38-25123 Brescia, Italy; lorenzo.montesano@unibs.it (L.M.); m.tocci@unibs.it (M.T.); marina.lavecchia@unibs.it (G.M.L.V.)

**Keywords:** cavitation erosion, ultrasound treatment, AlSi7, SEM

## Abstract

Ultrasound treatment of liquid aluminum alloys is known to improve mechanical properties of castings. Aluminum foundry alloys are frequently used for production of parts that undergo severe cavitation erosion phenomena during service. In this paper, the effect of the ultrasound treatment on cavitation erosion resistance of AlSi7 alloy was assessed and compared to that of conventionally cast samples. Cavitation erosion tests were performed according to ASTM G32 standard on as-cast and heat treated castings. The response of the alloy in each condition was investigated by measuring the mass loss as a function of cavitation time and by analyzing the damaged surfaces by means of optical and scanning electron microscope. It was pointed out that the ultrasound treatment increases the cavitation erosion resistance of the alloy, as a consequence of the higher chemical and microstructural homogeneity, the finer grains and primary particles and the refined structure of the eutectic induced by the treatment itself.

## 1. Introduction

Cavitation erosion phenomena are related to the rapid formation, growth, and collapse of bubbles due to the presence of strong pressure fluctuations in a liquid [1]. In a very short time, the bubbles collapsing produces shock waves on the surface of the material exposed to these micro-jets, which have a speed higher than 100 m/s [2]. When the pressure load exceeds the elastic limit of the material, this causes local plastic deformations able to induce pitting by dislodging particles from the surface itself [3,4]. The initial phase of the damaging phenomenon, called incubation time [5], does not involve any mass loss. However, the rapid repetition of the impact induces an accumulation of deformation until micro-failure occurs, with subsequent material loss [6]. In particular, it has been observed that the repetition of such pressure pulses on a solid surface can result in a fatigue-like failure [7,8].

Many authors investigated the behavior of different metals and alloys exposed to cavitation erosion during their working service. In particular, being frequently used for propeller applications, copper alloys have been widely analyzed [8,9,10,11,12]. Iron and steel, particularly stainless steel and cast iron, have been extensively studied for cavitation erosion resistance, especially with regard to the combined effect of a corrosive environment [13,14,15,16,17,18]. Similar investigations have been performed on Ti, Ni, and Co alloys [19,20,21,22,23,24].

Aluminum alloys are also frequently used for production of parts that undergo severe cavitation erosion phenomena during service**—**like diesel cylinder liners, hydrofoils, valves, sluice gates, etc. [25]. Typically, they are Al**–**Cu, Al**–**Mg, Al**–**Si**–**Mg or Al**–**Zn**–**Mg wrought alloys. These alloys have been widely studied [7,26,27], and also the synergic effect of aggressive fluids or slurries has been evaluated [28,29]. Concerning the aluminum foundry alloys, they are typically used for casting components like water and fuel pumps, cylinder blocks, pistons, heads and bases of combustion engines, propellers, valves, etc. [1,30]. Despite undergoing cavitation erosion damage, data about their behavior under these working conditions are less available in literature. Tomlinson et al. analyzed the effect of heat treatment on AlSi7 and AlSi11, finding a strong increase in erosion resistance due to age-hardening [31]. Other authors stated the poor behavior of AlSi as-cast alloys during cavitation erosion tests [32,33]. Some authors compared the cavitation erosion resistance of A356 alloy and A356FA5 composite containing 5 wt % fly ash, whose fine particles seem to suppress pit growth [34]. Additionally, similar investigations were also performed on composites reinforced with particles or fibers of silicon carbide and alumina [35,36].

More recently, some attempts have been made to correlate microstructural variations to cavitation erosion resistance for aluminum alloys [37]. As known, the application of ultrasonic treatment to liquid metals strongly affects the alloy microstructure, with changes from dendritic to almost globular, subsequently improving mechanical and corrosion resistance [38]. The idea of applying a dynamic action to a liquid metal to modify the final microstructure was mainly developed by D. Eskin [39]. As reported in literature, in fact, the introduction of high-power ultrasonic vibration into a liquid alloy leads to cavitation and acoustic streaming. Ultrasonic waves produce cavitation phenomena in liquids and the creation, growth, and collapse of bubbles, which can also generate very high impact forces. These have a dynamic role during nucleation because the high pressures fragment the rising crystals by breaking dendritic structures and by increasing the nucleation centers, while the acoustic flow induces a vigorous stirring of the bath, homogenizing the alloy [38]. Cavitation phenomena originate also a rapid development of hydrogen bubbles, causing their coalescence and flotation on the liquid metal surface and, therefore, promoting alloy degassing. Moreover, by means of ultrasound treatment, the wettability of non-soluble non-metallic particles**—**which are unavoidably present in the liquid metal**—**increases and heterogeneous nucleation is favored without the need of expensive inoculants [39]. Many authors have already demonstrated the effectiveness of this treatment on hydrogen removal [39,40], grain refinement [38,41], improved homogeneity of the casting [42], and preparation of feedstock material for semisolid processing [43], as an alternative to other conventional stirring methods [44].

To the authors’ knowledge, no data about the influence of microstructural variation promoted by ultrasound on cavitation erosion response of AlSi foundry alloys is available in literature. Hence, the aim of this paper is to investigate the effect of the ultrasound treatment of AlSi7, the most commonly used aluminum casting alloy, on the cavitation erosion resistance, comparing its behavior to the conventional cast alloy in both as-cast and age hardened conditions.

## 2. Experimental Procedure

The experiments were carried out on a hypoeutectic Al**–**Si alloy produced from commercially pure elements. No nucleant agents or modifiers were added. In Table 1, the chemical composition of the alloy, measured by GNR Metal Lab Plus spectrometer (GNR, Agrate Conturbia Novara**—**Italy), is reported.

About 1 kg of alloy was melted into an alumina crucible at 50 °C above the liquidus temperature, which is equal to 613 °C. A first set of samples was obtained by pouring the molten metal into cylindrical steel molds (26 mm in diameter and 50 mm in height) preheated at 150 °C.

These samples were named ‘NUST’, i.e., Not UltraSound Treated.

**Figure 1:**
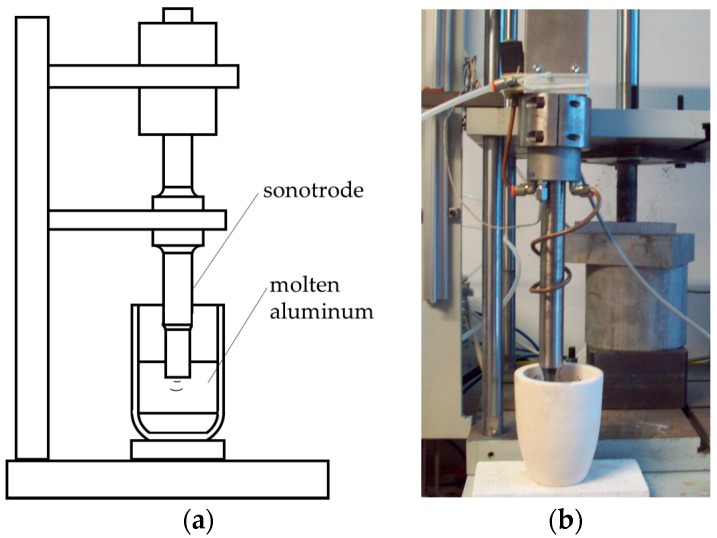
Sketch (**a**) and view of the of the ultrasound US apparatus (**b**) for the treatment of molten alloy.

A second set of samples, called ‘UST’ (UltraSound Treated), was produced by treating the alloy with ultrasounds (US) before being poured into the mold. In detail, the treatment was performed on the molten Al**–**Si alloy inside the crucible by means of a GV 2000 ultrasonic apparatus (Felisari, Verano Brianza**—**Milan, Italy), equipped with an Inconel 625 probe with final amplification diameter of 18 mm. Frequency, amplitude, and duration of the vibration were set at 20,000 ± 400 Hz, 80 µm, and 60 s respectively. In Figure 1, a sketch of the US treatment setup is shown, together with the real equipment.

Preliminary tests were performed in order to evaluate the possible dissolution of the probe into the molten aluminum alloy, with subsequent contaminations. To this aim, the US treatment was performed on more than 20 crucibles containing molten AlSi alloy and each corresponding chemical composition was measured by GNR Metal Lab Plus spectrometer. In all cases, the total amount of possible contaminants (i.e., Ni, Cr, Fe, Mo, and Nb) was lower than 300 ppm, therefore lower than the limits of the alloy according to the standard. Details of this study are reported elsewhere [45]. After demonstrating that no significant contamination of the molten metal is induced by UST, the investigation was focused on the analysis of the performance of the alloy.

A group of samples, in both NUST and UST conditions, were heat treated (T6) according to the following procedure: solution treatment for 4 h at 530 °C, quenching and artificial aging at 150 °C for 4 h. Therefore, four different alloy conditions were obtained.

From the cast cylinders, 10 mm thick disks were machined. One base surface of the disks was ground and polished up to a mirror finish with standard metallographic techniques to allow microstructure analysis and cavitation-erosion tests. Regarding the surface preparation for hardness tests, grinding operations were performed with up to 320 grid paper.

The microstructure was assessed by a Leica DMI3000 optical microscope (Leica Microsystems, Wetzlar, Germany) equipped with a Leica digital camera (Leica Microsystems, Wetzlar, Germany). Grain size measurements were carried out in polarized light. Before the observations, samples were subjected to an electrochemical etching in HBF_4_ 3 wt % solution with an applied bias of 20 V. Grains were identified with chromatic variation and their dimension was assessed by the intercept method defined in the NF A04**–**503 standard [46].

Brinell hardness tests were performed by a Galileo Ergotest Comp25 apparatus (LTF Galileo Italy), with an applied load of 62.5 kg and an indenter with a radius of 2.5 mm. Reported values are the average of at least five indentations per sample.

Cavitation-erosion investigations were carried out according to ASTM G 32 standard, applying the “stationary specimen method” [47] and using the same Felisari GV 2000 equipment at the same frequency (20 kHz) but changing the amplitude to 50 µm. As reported in the standard, this test method produces cavitation damage on the face of a specimen under high frequency vibration, while immersed in a liquid. The vibration induces the formation and collapse of bubbles in the liquid, which in turn produces the erosion of the specimen, detectable as material loss. The standard stated that, although the mechanism for generating fluid cavitation in this method differs from that occurring in real systems, the damaging mechanism is believed to be basically similar. Hence, the method represents a small-scale test that enables both comparison of the cavitation erosion resistance of different materials and study of the nature and evolution of the damage [47].

**Figure 2:**
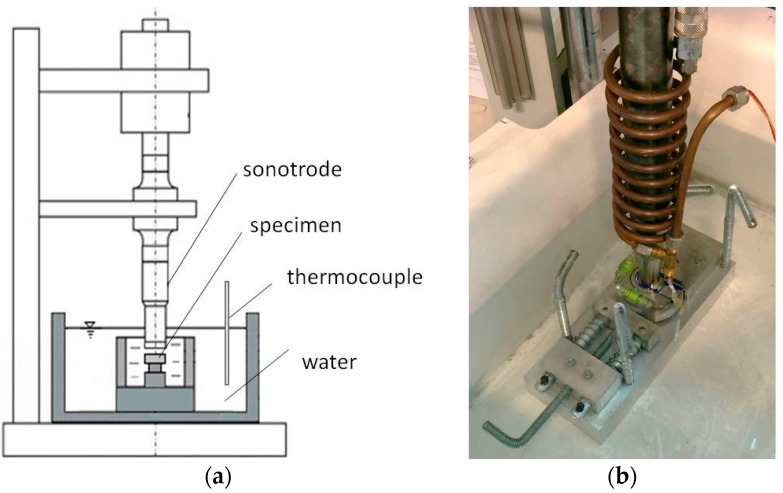
Sketch of the US apparatus (**a**) and view of the holding system (**b**).

Thus, in agreement with the standard, the specimen was immersed in distilled water and the vibrating tip of the horn was placed in close proximity to it. In this way, the cavitation bubbles induced by the horn acted on the specimen. In the used configuration, the horn tip had a diameter of 18 mm, thus the exposed area resulted about 254 mm^2^. The specimens were fixed in a holding system at 0.5 mm from the horn tip. As shown in Figure 2, both the holding system and horn were placed in a tank filled with distilled water at constant temperature (room temperature).

Based on the standard rules, the test specimen has to be weighed accurately before testing begins and again during periodic interruptions of the test, in order to obtain a history of mass loss versus time. Appropriate interpretation of this cumulative erosion versus-time curve permits comparison of results between different materials. Hence, at first, short tests were performed (0.5, 1, 2, 4, 8, 12, 24, 36, 48, and 60 min) in order to analyze the cavitation mechanism and to point out the differences induced by the modification of the microstructure from NUST to UST in the early stage of the damaging. Then the experiments were prolonged up to 8 h, according to the standard. In particular, sample damaging was monitored every hour during the tests, measuring the mass loss of the specimens with a precision balance having a sensitivity of 0.1 mg.

The eroded surfaces, after each test interruption, were analyzed by means of Scanning Electron Microscope (SEM), LEO EVO 40 (Carl Zeiss AG, Milan, Italy).

## 3. Results and Discussion

**Figure 3:**
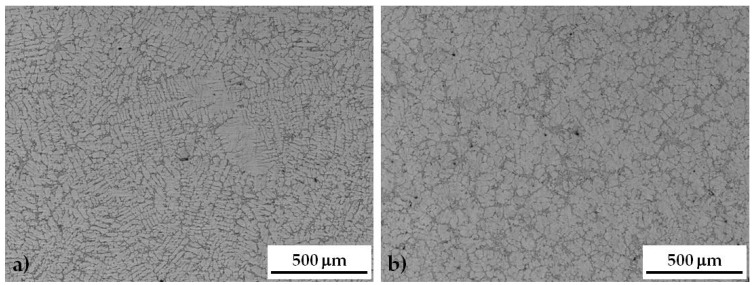
Optical microscope image of not ultrasound treated NUST (**a**) and ultrasound treated UST (**b**) sample microstructure.

In Figure 3a, comparison between the microstructure of as-cast NUST and UST samples is reported. NUST samples show the typical microstructure of casting products, characterized by the dendritic Al-rich phase (light gray in the images) surrounded by the eutectic, where some micro-shrinkage porosity can be detected (Figure 3a). The application of ultrasound to the liquid alloy produced a finer microstructure with an almost globular or rosette type morphology of the primary Al-rich phase (Figure 3b). It was found that, after US treatment, grain size decreases from 501 ± 115 μm for the NUST sample to 210 ± 61 μm for the UST sample, confirming the effectiveness of US treatment in reducing grain size, as already demonstrated for wrought alloys in a previous work by the authors [37]. This effect is due to the multiplication of solidification nuclei by activating heterogeneous insoluble substrates (such as oxides, always present in liquid aluminum) [38,39]. In fact, it is known that nonmetallic inclusions**—**like oxides usually dispersed**—**serve as hydrogen concentrators in liquid aluminum alloys. This makes these particles efficient cavitation nuclei and decreases the cavitation threshold [39]. As discussed in literature, in metallic melts, where free gas bubbles are hardly possible, only solid nonmetallic inclusions may qualify as cavitation nuclei because gas can exist in capillaries on the surface of the inclusions. In real melts containing oxides or other insoluble particles, hydrogen precipitates on poorly wetted insoluble inclusions and, generally, the precipitation follows the basic rules of heterogeneous nucleation and growth [39].

**Figure 4:**
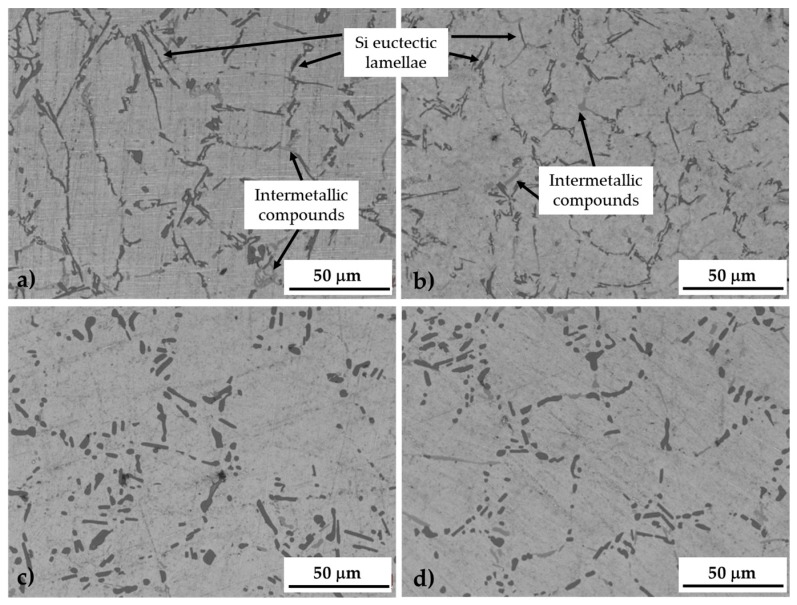
Microstructure of as-cast NUST (**a**) and UST (**b**) samples and heat treated T6 NUST (**c**) and UST (**d**) samples.

**Figure 5:**
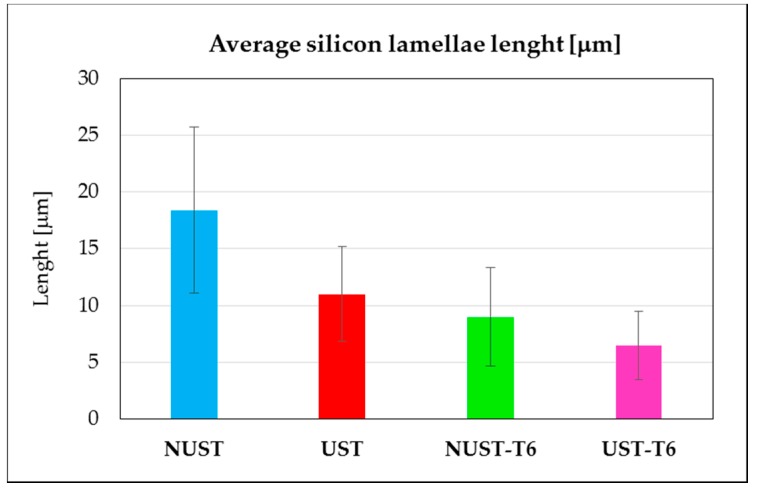
Average length of silicon lamellae.

At higher magnification, it can be noted that the US treatment slightly induced a refining effect on the silicon eutectic lamellae as well as on intermetallic compounds (Figure 4a,b) [38,39]. As already stated by Puga et al., in fact, cavitation bubbles induced by UST can behave like atoms of chemical modifiers, promoting the modification of silicon. Additionally, the decrease of temperature at the bubble/melt interface during the expansion phase of a cavitating bubble suggests that the treatment can also influence the nucleation frequency of eutectic cells, promoting the nucleation of a higher number of silicon particles [48]. As a result, UST alloy presented an average length of the Si lamellae equal to 10.6 μm vs 18.4 μm for the NUST one. Additionally, the standard deviation is reduced from 7.3 μm for the NUST to 4.2 μm for the UST. These differences resulted more limited after the solution treatment (4.36 μm for the NUST vs 3 μm for the UST samples), which corresponds to the first phase of the T6 treatment, able to modify the size and morphology of the eutectic silicon. The induced spheroidization and coarsening of the Si particles can be clearly noted in (Figure 4c,d). In Figure 5, the average length of silicon lamellae in the different samples is summarized.

The average hardness values for the four samples, with the respective standard deviations, are listed in Table 2. It can be noted that the alloy in NUST condition is softer than in the other ones. The light improvement of hardness in UST samples can be related to the finer grains induced by the treatment [39]. The smaller standard deviation in both grain size and hardness measurements can be due to the homogenizing effect of the ultrasound itself, which also promotes a more uniform microstructure and solute distribution, as already demonstrated by the authors elsewhere [38,42] as well as being known from literature [39,49,50,51].

Some authors report the hardness as an indicator of the erosion resistance, in particular the higher the hardness, the higher the cavitation resistance [7,52]. Therefore, the hardness of the alloys was measured in order to define the eventual correlation between this mechanical property and the resistance to damage. Based on this, the NUST sample can be expected to exploit almost the same performances as the UST one, in as-cast and T6 conditions respectively.

**Figure 6:**
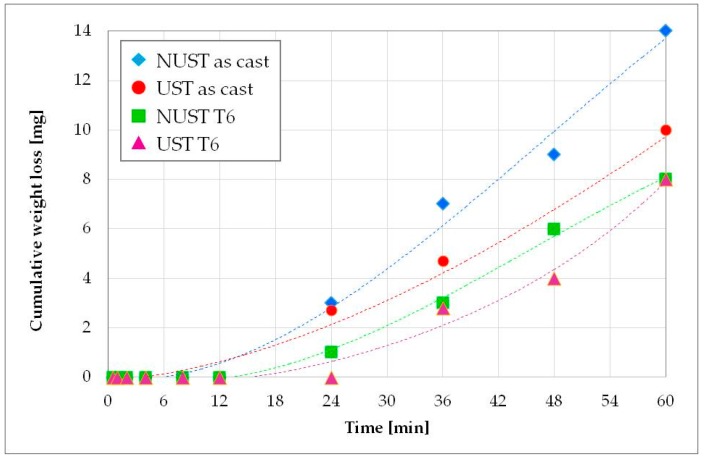
Incubation period.

At first, the incubation period was estimated, i.e., the initial stage of the damaging phenomenon. During this stage, the erosion rate is negligible and it represents the accumulation of plastic deformation and internal stresses under the surface, which precedes significant material loss [47]. According to the ASTM G32 standard, the incubation period is quantitatively defined as the intercept, on the time axis, of a straight-line extension of the maximum-slope portion of the cumulative erosion-time curve [47]. Thus, the cumulative mass loss curves vs time for the different alloy conditions during the first hour of exposure were plotted, as shown in Figure 6. By the intercepts to these curves, the incubation time results in the order of 15 min for both the as-cast UST and NUST alloys, slightly higher for the NUST T6 sample (about 19 min) and about double (27 min) for UST T6 one. It follows that a general trend of higher incubation time with increasing hardness can be highlighted. However, a direct proportionality cannot be stated. In fact, analyzing the evolution of the damage (Figure 6), the UST as-cast alloy seems to react more closely to NUST T6 than NUST as-cast, notwithstanding its hardness is substantially lower than that of NUST T6 alloy but comparable to that of the as-cast NUST. This result can be related to the higher chemical homogeneity, the refinement of the grains and primary particles, their uniform distribution in the volume, and the refined or modified structure of the eutectic after the ultrasound treatment [39,48,49].

**Figure 7:**
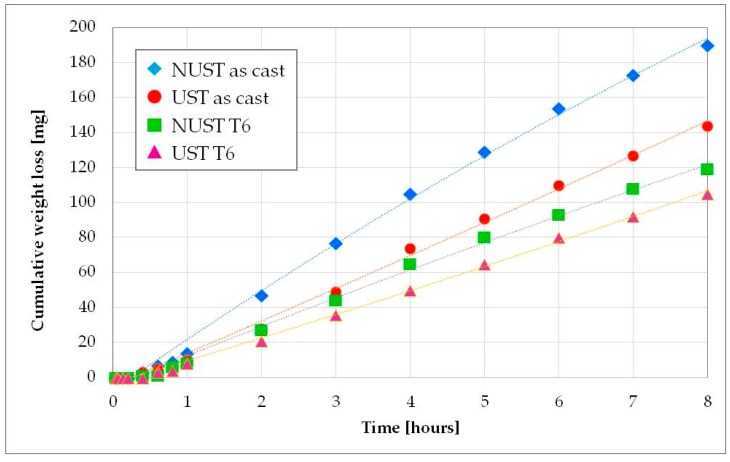
Cumulative mass loss vs time.

After the incubation phase, the cumulative mass loss increases almost linearly in all the tested conditions (Figure 7). As expected, the heat-treated alloys showed improved performances, similarly to other Al alloys [1,7,32]. Increasing the time, the worse behavior of the NUST as-cast alloy compared to the cast UST one appears more evident. This confirms that the modification of the microstructure induced by the US treatment strongly affects the cavitation erosion resistance. In fact, although NUST and UST as-cast alloys showed similar hardness values, the weight loss of the dendritic microstructure resulted about 32% higher than that of the US one. Furthermore, the linear interpolation of the experimental points was carried out and the erosion rate was evaluated as the slope of the linear curves (Table 3). It can be noted that the erosion rate of the NUST as-cast alloy is 35% higher than that of the UST as-cast one.

The age hardening treatment increases the mechanical resistance of the alloys and also the cavitation erosion resistance. Again, the dendritic microstructure showed a worse behavior, with an erosion rate and weight loss 15.6% and 13.3% respectively higher than the corresponding UST T6 microstructure. The smaller difference, in comparison with the as-cast condition, can be explained by analyzing the microstructure of the two samples (Figure 4c,d). As mentioned above, in fact, the heat treatment induces a spheroidization of the eutectic silicon particles. Additionally, because of the maintenance at high temperature during the solution treatment, a homogeneous solid solution is formed when atoms leave the coarse particles formed during solidification and diffuse into the matrix, reducing the concentration gradient [53]. This homogenizing effect results in a diminished difference between NUST and UST microstructure and, thus, cavitation erosion resistance.

As previously observed for incubation time, also for the erosion rate, a general trend of higher resistance with increasing hardness can be highlighted but a linear correlation cannot be stated, which is in agreement with other authors [7,26,31,52].

**Figure 8:**
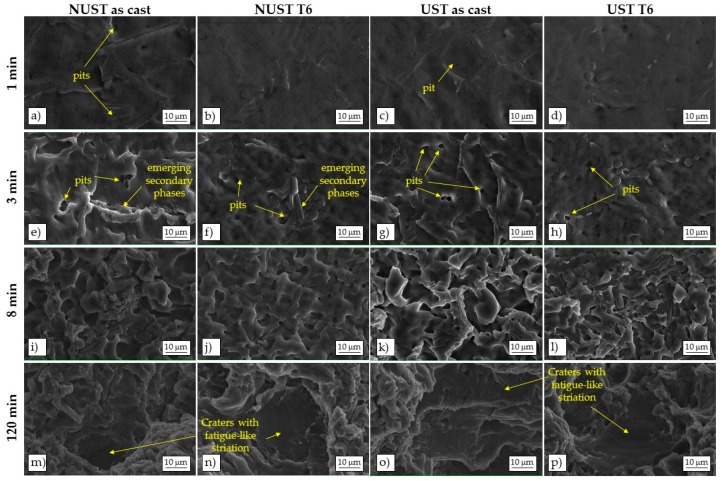
Surface topographies of the damaged areas of NUST as-cast (**a**,**e**,**i**,**m**); NUST after T6 (**b**,**f**,**j,n**); UST as-cast (**c**,**g**,**k,o**); and UST after T6 (**d**,**h**,**l,p**).

The eroded surfaces of all the samples were examined by SEM analysis at different intervals during the incubation period as well as after longer exposure to cavitation, in order to identify the origin and evolution of the damage. A selection of the collected images is shown in Figure 8.

The primary Al-rich phase appears to be the primary site for cavitation attack at early stage of erosion. Small pits can be frequently detected already after 1 min of exposure in as-cast NUST and UST alloys, probably due to individual bubbles acting on the surface of the metal (Figure 8a**–**d) [3].

After 3 min (Figure 8e**–**h), the number of pits increased and undulations and plastic deformation of the surface can be seen, particularly in the as-cast alloys. The more massive deformation is produced by the impact pressure of many thousands of bubbles collapsing together [3] that induce a localized temperature increase (in the order of hundreds of centigrade degrees) [54] and reduce the strength of the metal surface, enhancing the damage. In the case of dendritic microstructures, secondary phases**—**as Si particles and intermetallic compounds**—**seem to arise on the damaged surface (Figure 8e,f) because of the deformation and removal of the surrounding Al-rich phase matrix. Therefore, the damage can follow the dendritic network. On the contrary, in the UST microstructure, the primary phase globules are less interconnected and the secondary phases, better distributed along their boundaries, seem to offer a barrier to the propagation of the damage, providing an anchor to the underlying material and resisting detachment [31].

At advanced stages, for instance after 8 min of exposure (Figure 8i**–**l), the progress of cavitation damage did not remain limited to the primary Al-rich phase. The same ductile fracture mechanism, with ductile shearing of the protruding rims of the craters, can be recognized in all the alloys, although it is more pronounced on as-cast ones.

The analysis of the eroded areas after 2 h of exposure (Figure 8m**–**p) shows the same damage mechanism for all the alloys. More in detail, as-cast NUST surface presents deeper craters (Figure 8m) than the others (Figure 8n**–**p). Some areas with smooth surface are also present (Figure 8m**–**p), characterized by fatigue like fracture with striated and flat bottom, caused by the cyclic oscillation of the pressure during the test [31].

**Figure 9:**
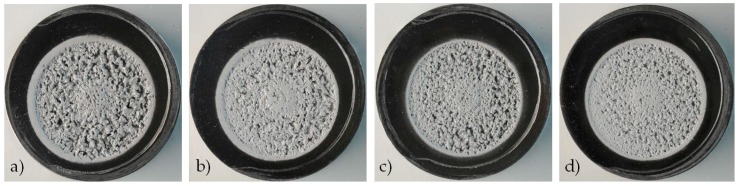
Surface topographies of the damaged areas of NUST as-cast (**a**); NUST after T6 (**b**); UST as-cast (**c**); and UST after T6 (**d**).

Figure 9 exhibits a comparison of the eroded area, at macroscopic scale, of the four specimens after 8 h of exposure. It can be clearly seen that the strongest damage was experienced by the NUST alloy in the as-cast condition (Figure 9a), whereas the highest erosion resistance was exhibited by the heat-treated UST sample (Figure 9d). The damage of the NUST after T6 appears quite similar to that of as-cast UST sample (Figure 9b,c).

**Figure 10:**
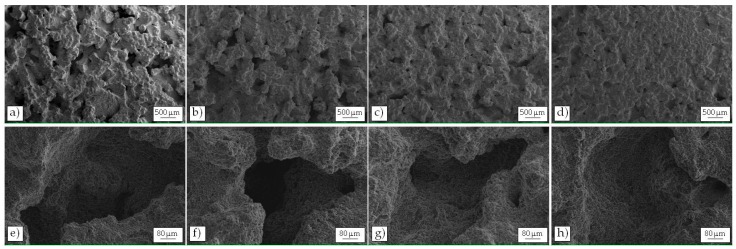
SEM micrographs at different magnification of the eroded surface of NUST as-cast (**a**,**e**); NUST after T6 (**b**,**f**); UST as-cast (**c**,**g**); and UST after T6 (**d**,**h**) samples.

At higher magnification, the eroded surface of the NUST as-cast alloy (Figure 10a**–**e) showed a larger number of pits and wider cracks (in the order of 700 µm) than the other tested conditions (Figure 10b**–**d,f**–**h). In particular, NUST T6 (Figure 10b,f) and UST as-cast (Figure 10c,g) revealed similar damage, with a slightly higher amount of pits in the UST as-cast sample. The same damaging mechanism was shown by the UST T6, whose higher resistance is demonstrated by the lower amount of pits that seem less deep (Figure 10d,h).

**Figure 11:**
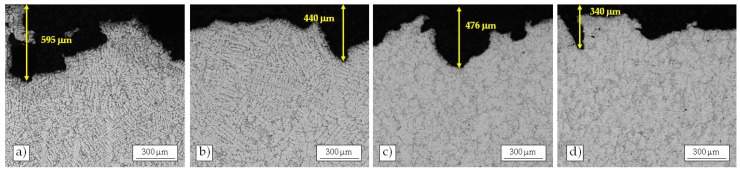
Cross-sectional micrographs of NUST as-cast (**a**) and NUST T6 (**b**); UST as-cast (**c**); and UST T6 (**d**) samples near severe erosion regions after 8 h of cavitation erosion.

To deeply analyze the damaging mechanism, metallographic analyses were carried out on the cross section of the eroded areas after 8 h of exposure (Figure 11). Low magnification images were collected with the upper edge of the picture coincident with the undamaged samples surface, in order to better discern the magnitude of the damage depth.

All the alloys show pits or craters. However, they resulted more pronounced on the NUST surface than on the as-cast UST one, confirming the previous observations. In particular, the depth of one of the deepest erosion pits detected on the as-cast NUST sample was 595 µm, while that of the deepest erosion pits observed on the same alloy after ultrasound treatment is in the order of 476 µm (i.e., around 20% smaller). The same trend was observed for the NUST and UST alloy after heat treatment, whose deepest pits were around 440 µm and 340 µm, respectively. The analysis on the cross sections of the eroded areas further confirm the previous finding. In fact, it shows the improvement on cavitation erosion resistance induced by the US treatment, so that the as-cast UST alloy behaves similarly to the NUST T6.

## 4. Conclusions

The effect of ultrasound treatment on the cavitation erosion resistance of an AlSi7 alloy has been investigated.

The analysis of the eroded areas showed the same damaging mechanism for all the alloy conditions. However, it was found that the ultrasound treatment of liquid metal enhances material resistance to cavitation phenomena, as visible from the measurements of weight loss as a function of time after several hours of exposure to cavitation. It was established that this is mainly due to the modified globular microstructure of UST samples, which is characterized by smaller grain size, a less interconnected primary phase than the dendritic one, a finer eutectic phase and a more uniform distribution of solute and secondary phases. This created a barrier against the propagation of the erosion mechanism, which started from the soft aluminum matrix, as observed by SEM analysis of the specimen surface after different exposure times. As a consequence, UST samples exhibited a reduced weight loss in comparison with NUST ones. Heat treatment lead to an increase in material hardness and cavitation resistance for both NUST and UST conditions, but did not affect the erosion mechanism and confirmed the better performance of UST samples than NUST ones, as additionally supported by the observation of the cross section of the eroded samples.

These findings can be helpful in defining an effective treatment in order to improve the cavitation resistance of foundry alloys.

## Figures and Tables

**Table 1:** Chemical composition (wt %) of the investigated Al**–**Si alloy.

Alloy	Designation	Si	Mg	Fe	Ti	Cu, Zn, Mn, Ni, Cr	Al
AlSi7	A356	7.123	0.33	0.0191	0.0015	<0.02%	Balance

**Table 2:** Hardness measurements.

Sample Name	Mean HB	St. Dev.
NUST	66.8	1.0
UST	71	0.8
NUST T6	107.9	3.1
UST T6	110.9	1.6

**Table 3:** Slope of the cumulative mass loss curves.

Sample Name	Slope of the Curve (mg/min)
NUST	25.88
UST	19.15
NUST T6	16.24
UST T6	14.05
